# Thrombotic Complications after COVID-19 Vaccination: Diagnosis and Treatment Options

**DOI:** 10.3390/biomedicines10061246

**Published:** 2022-05-26

**Authors:** Katharina Guetl, Reinhard Bernd Raggam, Thomas Gary

**Affiliations:** Division of Angiology, Department of Internal Medicine, Medical University of Graz, 08036 Graz, Austria; reinhard.raggam@medunigraz.at (R.B.R.); thomas.gary@medunigraz.at (T.G.)

**Keywords:** thrombosis, vaccination, adenoviral vector vaccines, anticoagulation, (autoimmune) heparin-induced thrombocytopenia (HIT), thrombosis thrombocytopenia syndrome (TTS), vaccine-induced thrombotic thrombocytopenia (VITT), severe acute respiratory syndrome coronavirus 2 (SARS-CoV-2), coronavirus disease 2019 (COVID-19)

## Abstract

Coronavirus disease 2019 (COVID-19) vaccines were developed a few months after the emergence of the pandemic. The first cases of vaccine-induced thrombotic complications after the use of adenoviral vector vaccines ChAdOx1 nCoV-19 by AstraZeneca, and Ad26.COV2.S by Johnson & Johnson/Janssen, were announced shortly after the initiation of a global vaccination program. In these cases, the occurrence of thrombotic events at unusual sites—predominantly located in the venous vascular system—in association with concomitant thrombocytopenia were observed. Since this new entity termed vaccine-induced thrombotic thrombocytopenia (VITT) shows similar pathophysiologic mechanisms as heparin-induced thrombocytopenia (HIT), including the presence of antibodies against heparin/platelet factor 4 (PF4), standard routine treatment for thrombotic events—arterial or venous—are not appropriate and may also cause severe harm in affected patients. Thrombotic complications were also rarely documented after vaccination with mRNA vaccines, but a typical VITT phenomenon has, to date, not been established for these vaccines. The aim of this review is to give a concise and feasible overview of diagnostic and therapeutic strategies in COVID-19 vaccine-induced thrombotic complications.

## 1. Introduction and General Aspects

Coronavirus disease 2019 (COVID-19), as caused by severe acute respiratory syndrome coronavirus 2 (SARS-CoV-2), was first reported in Wuhan, China, in December 2019. The rapid spread of COVID-19 throughout the world led to the declaration of a pandemic, which is still ongoing. Since potential treatments are still undergoing evaluation, the development of vaccines against SARS-CoV-2 remains the most important countermeasure in order to halt the pandemic. Within the time period from December 2020 to March 2021, a total of four vaccines based on two different mechanisms of action were approved by the European Medicines Agency (EMA). While the two messenger RNA-based vaccines BNT162b2 (Pfizer-BioNTech, New York, NY, USA; Mainz, Germany) and mRNA-1273 (Moderna, Cambridge, MA, USA) act via encoding of a SARS-CoV-2 spike protein, ChAdOx1 nCoV-19 (AstraZeneca, Cambridge, UK) and Ad26.COV2.S (Johnson & Johnson/Janssen, New Brunswick, NJ, USA) are recombinant adenoviral vectors encoding SARS-CoV-2 spike glycoprotein [1]. Over the first few weeks after the initiation of global vaccination programs, an accumulation of thrombotic events, predominantly at unusual sites, frequently in combination with thrombocytopenia in otherwise healthy individuals, and following recombinant adenoviral vector vaccine administration, was observed. Scientific workup of this new entity, called vaccine-induced thrombotic thrombocytopenia (VITT), unveiled similarities between these cases and heparin-induced thrombocytopenia (HIT) by the presence of functional antibodies against platelet factor 4 (PF4) in the absence of heparin [2,3,4,5,6]. For this reason, the conclusion was drawn that COVID-19 adenoviral vector vaccines may cause the rare complication of immune thrombotic thrombocytopenia, resembling the clinical picture of autoimmune HIT [2]. Autoimmune HIT indicates the presence of antibodies directed against a PF4-polianion complex, which are able to activate platelets without prior exposure to heparins [7]. Apart from “VITT”, other terms have been adopted, including vaccine-induced prothrombotic immune thrombocytopenia (VIPIT), thrombosis with thrombocytopenia syndrome (TTS), or vaccine-induced immune thrombotic thrombocytopenia (VIITT), all of which describe the same phenomenon observed after COVID-19 vaccination [8]. Cerebral venous thrombosis (CVT), a distinct cerebrovascular disorder, is potentially the most serious form of VITT. CVT encompasses thrombosis in dural sinus veins, cortical veins, and also in deep venous structures. Venous clots typically develop in a dural sinus, but may propagate to cortical veins. Isolated cortical vein thrombosis is a very rare condition [9]. CVT most commonly affects young women with underlying prothrombotic risk factors [10]. Already in 2005, HIT was known as a risk factor for the development of CVT [11]. Strictly speaking, the term ‘cerebral venous sinus thrombosis’ (CVST) is limited to thrombotic events located in dural sinus veins, but it is also commonly used in literature instead of CVT. The authors decided for a consistent use of CVT within this present manuscript. While the incidence of CVT in the general population is estimated to occur at a maximum of 2.0 cases/100,000 people per year [12], the cumulative incidence of CVST following vaccination with COVID-19 adenoviral vector vaccines ranges from 0.32 up to 6.5 cases per 100,000 vaccinated individuals [3,6,13,14,15]. The EMA reports CVT in the context of VITT to appear most frequently in women aged below 60 years [16,17]. Mortality rates for thrombotic events following COVID-19 vaccination are reported to be up to 25%, and are thus two-to-three times higher as compared to non-vaccine-induced thrombotic events [8]. The emergence of the VITT phenomenon as a severe and potentially life-threatening complication post-COVID-19 vaccination using adenoviral vector vaccines has caused governmental restrictions and also temporary suspensions of these vaccines. Referring to pathophysiological knowledge, non-heparins are recommended for anticoagulant treatment in these patients. Apart from anticoagulant treatment, administration of high-dose immunoglobulins (IVIG) plays an important role in VITT therapy by causing interruption of the prothrombotic pathomechanism behind VITT and also effectively inhibiting platelets [2]. To date, the World Health Organization (WHO) reports approximately 520 million confirmed cases and more than 6 million deaths in association with COVID-19, but also the administration of more than 11 billion vaccine doses [18]. mRNA vaccines by Pfizer-BioNTech and Moderna have been procured globally 2.5 times more often than the recombinant adenoviral vector vaccines produced by AstraZeneca and Johnson & Johnson/Janssen. In total, 44% of all globally procured doses among those four aforementioned vaccines were produced by Pfizer-BioNTech [19]. Current European data show that a total of 787,920,773 mRNA vaccine doses (Pfizer-BioNTech: 633,033,372; Moderna: 150,887,401) and 88,819,460 adenoviral vector vaccine doses (AstraZeneca: 69,158,774; Janssen: 19,660,686) have been administered [20]. By 19 May 2022, 65.7% of the world’s population had received at least one dose of a COVID-19 vaccine [21].

The aim of this review is to give an overview of (1) authorized COVID-19 vaccines, (2) thrombotic complications following a COVID-19 vaccination, (3) the pathomechanism behind the new phenomenon of vaccine-induced thrombotic thrombocytopenia (VITT), and (4) diagnostic and treatment recommendations for VITT.

## 2. COVID-19 Vaccines

### 2.1. Vaccine Development and Approval

The development of vaccines against SARS-CoV-2—from the release of the genome sequence until the approval of several vaccines for public use—took more or less one year. This fast-tracked development became desirable due to global losses in human resources and in the economy. Various strategies were explored worldwide in the search for safe and effective vaccines, including DNA vaccines, mRNA vaccines, protein subunit vaccines, recombinant viral vector vaccines, live attenuated vaccines, whole killed vaccines, and virus-like particle vaccines [22]. By January 2022, a total of eight vaccines had been listed for emergency use by the WHO: ChAdOx1 nCoV-19 (AstraZeneca), Ad26.COV2.2 (Johnson & Johnson/Janssen), BNT162b2 (Pfizer-BioNTech), mRNA-1273 (Moderna), BBIBP-CorV (Sinopharm, Beijing, China), CoronoVac (Sinovac, Beijing, China), Nuvaxovid/Covovax NVX-CoV2373 (Novavax, Gaithersburg, MD, USA), and BBV152 COVAXIN (Bharat Biotech, Hyderabad, India) [23]. In contrast, five COVID-19 vaccines have been authorized by the EMA for use in the European Union (EU): ChAdOx1 nCoV-19 (AstraZeneca), Ad26.COV2.2 (Johnson & Johnson/Janssen), BNT162b2 (Pfizer-BioNTech), mRNA-1273 (Moderna), and Nuvaxovid (Novovax). COVID-19 mRNA vaccines and recombinant adenovirus vector vaccines represent the most frequently used vaccines in the EU. Some more vaccines are currently under review or awaiting market authorization [24].

### 2.2. mRNA Vaccines

COVID-19 mRNA vaccines authorized by the EMA comprise BNT162b2 (Pfizer-BioNTech) and mRNA-1273 (Moderna). The mechanism of action of mRNA vaccines is the delivery of short modified viral mRNA sequences in order to express a mutated form of a SARS-CoV-2 protein inside the host cell, reaching a comparable immunogenicity as with inactivated virus materials. Since naked mRNA is highly susceptible to ribonuclease, lipidic nanoparticles encase mRNA sequences to enable stable packaging and also to facilitate mRNA delivery, overcoming physiological barriers [25]. mRNA vaccines do not only stimulate adaptive immunity by antibody production, but also cause T-cell responses within the spectrum of innate immune responses [26,27]. Repeated administration of mRNA vaccines at certain intervals is required due to transient antigenic expression. Host genetic expression is not affected, since these mRNA vaccines do not interfere with genetic recombination [25]. The BNT162b2 vaccine by Pfizer-BioNTech was the first vaccine approved by the WHO and the EMA by end of December 2020. General recommendations comprise a two-dose schedule, each at 30 µg, administered intramuscularly and three to four weeks apart [28]. The mRNA-1273 vaccine by Moderna was authorized by the EMA in January 2021, and requires intramuscular administration of two doses 28 to 42 days apart [29].

### 2.3. Recombinant Viral Vector Vaccines

The mechanism of action of the recombinant viral vector vaccines ChAdOx1 nCoV-19 (AstraZeneca) and Ad26.COV2.2 (Johnson & Johnson/Janssen) is based on SARS-CoV-2 protein production in host cells. Viral vectors are mandatory in order to enable access to human cells. Non-human viruses or rare serotype viruses are selected as vector viruses to prevent influence on pre-existing host immunity [30]. The viral vector vaccine ChAdOx1 nCoV-19 by AstraZeneca is a chimpanzee adenovirus DNA vector encoding the S glycoprotein of SARS-CoV-2. Two separate doses, four to twelve weeks apart, are recommended [31,32]. Ad26.COV2.S is a recombinant but non-replicating human adenovirus encoding SARS-CoV-2 protein. A single intramuscular injection is sufficient for this vaccine [33].

## 3. Thrombotic Complications after COVID-19 Vaccination

By end of February 2021, only a few weeks after the initiation of global vaccination programs, several cases of thrombosis at unusual sites had been reported. Greinacher et al. [2] summarized observations on 11 patients from Germany and Austria in whom a diagnosis of thrombosis or thrombocytopenia after vaccination with the AstraZeneca adenovirus vector vaccine ChAdOx1 nCov-19 was made. Of those 11 patients, 9 were women aged less than 50 years. Approximately half of the entire of thrombotic events diagnosed were located in the cerebral veins (9/19, 47.4%), and splanchnic vein thrombosis and pulmonary embolism were each detected in three patients (3/19, 15.8%). Interestingly, a total of 19 thrombotic events were identified in only 11 patients, leading to the conclusion of a frequent appearance of multilocular thrombosis. Symptom onset was documented between five and sixteen days after the application of a first shot of the ChAdOx1 nCoV-19 vaccine. All patients presented with concomitant moderate to severe thrombocytopenia, raising the question of a clinical resemblance of this new entity to HIT. For this reason, serum obtained was analyzed by use of a standard enzyme-linked immunosorbent assay (ELISA) for the detection of PF4-heparin antibodies, and a PF4-dependent platelet activation test for functional analyses. Evidence of disseminated intravascular coagulation (DIC) by means of a massive increase in D-Dimer levels, abnormalities in routine coagulation monitoring parameters, and fibrinogen levels, was found in 45% of patients [2]. In the United States (US), six cases of CVT together with thrombocytopenia, following administration of approximately 7 million Ad26.COV2.S vaccine doses, resulted in a temporary nationwide suspension of Ad26.COV2.S vaccinations in April 2021. Together with six more CVT cases, a case series of 12 patients who developed CVT and thrombocytopenia after receiving a Ad26.COV2.S vaccination has been published. A total of 11 patients were tested for the presence of antibodies against heparin-PF4, all with positive results. All patients were females younger than 60 years of age, who experienced an onset of symptoms by day six post-vaccination at the earliest, and by day fifteen post-vaccination at the latest [5]. Additional cases and/or case series on VITT patients were published by Schultz et al. [3], Franchini et al. [34], and Scully et al. [4]. In total, the five publications mentioned report on 79 thrombotic events in 52 patients. Table 1 summarizes those reported VITT cases. Testing for anti-PF4 antibodies was performed on 48 patients and antibody presence was confirmed in 92.3%. Regarding outcomes, 20 out of 52 patients (38.5%) died. Patients were aged between 21 and 77 years, with a proportion of 88.5% below the age of 60 years. 75% were females, corresponding to a gender ratio of 3:1 female:male [35]. By November 2021, an extensive analysis of 213 cases of CVT following SARS-CoV-2 vaccination reported to the EMA was published. Of the cases identified, 87.8% occurred post-vaccination with the ChAdOx1 nCoV-19 vaccine, but the remaining 12.2 % were detected after mRNA vaccination, all after the BNT162b2 (Pfizer-BioNTech) vaccination, except for one after the mRNA-1273 (Moderna) vaccination. Thrombocytopenia was found in the majority of patients who received the ChAdOx1nCoV-19 vaccine (107/187, 52.2%), but in none who received the mRNA vaccination. Since there was no case reported to the EMA of CVT following the Ad26.COV2.S vaccination during the study period from 20 December 2020 to 8 April 2021, the Ad26.COV2.S vaccine was not further considered in the aforementioned analysis [36]. Overall, the complete image of autoimmune HIT was fulfilled by observations of thrombotic events with concomitant thrombocytopenia, and the detection of platelet activating antibodies against PF4 in the absence of heparin. Available evidence on prothrombotic disorders resembling HIT but occurring in the absence of heparin have been described in the past decades in association with the application of polyanionic drugs, viral and bacterial infections, and knee-replacement surgery [7,37,38,39,40,41,42,43]. Aside from the complete image of VITT, vaccine-induced thrombocytopenia—so called VIT—may occur as a preceding syndrome, without any manifest thrombotic event. Propagation to VITT might be prevented by the immediate implementation of therapeutic measures as recommended for VITT. A case series including 11 patients reports on the occurrence of severe headaches presenting with typical VITT laboratory findings, including thrombocytopenia, high D-Dimer levels, and high titers of antibodies directed against PF4, from five to eighteen days after vaccination with ChAdOx1 nCov-19, but in the absence of morphologically detectable CVT [44].

## 4. The Pathogenesis of Vaccine-Induced Thrombotic Thrombocytopenia

Thrombotic thrombocytopenia following COVID-19 adenoviral vector vaccines mimics the clinical image of autoimmune HIT by showing strong reactivity on a PF4-heparin enzyme-linked immunosorbent assay (ELISA), due to high-titer immunoglobulin G class antibodies directed against PF4 with enhanced platelet activation in the presence of PF4, but also inhibition, rather than activation, when adding low molecular weight heparin (LMWH) [2,3,7]. The enhancement of platelet activation by PF4 has been designated a typical sign of HIT [45,46]. Available evidence also describes interaction between adenovirus and platelets, and even platelet activation by adenovirus [47,48]. In HIT, PF4 is enriched at the vessel wall and locally released following platelet activation [49,50]. Further scientific research into the immunopathogenesis behind the VITT syndrome visualized the complex formation of ChAdOx1 nCov-19 vaccine components with PF4 on platelet surfaces, and the binding of anti-heparin/PF4 antibodies to these antigenic complexes. A two-step mechanism underpins the processes of the VITT phenomenon: the first “early” step at days one to two following vaccination is characterized by the complex formation of vaccine components with PF4, but also further stimulation of proinflammatory immune responses due to systemic dissemination of the vaccine components. These proinflammatory processes act as triggers in the amplification of anti-PF4 antibody production. In the second “late” step, between days five and twenty after vaccination, antibodies directed against PF4, resembling those in autoimmune HIT, activate platelets in a PF4- and polyanion-dependent manner. Apart from this, VITT antibodies against PF4 also activate granulocytes to release neutrophil extracellular traps (NETs) in the presence of platelets, and are dependent on PF4. The fragmentation of NETs by DNAses allows amplification of platelet activation due to extracellular circulating DNA [51]. This two-step mechanism that underpins the development of thrombotic events in VITT patients strictly resembles the pathomechanism of autoimmune HIT and also of classic HIT. Anti-PF4/heparin antibodies are frequently observed following heparin treatment, but only a small proportion of patients develop the clinical picture of HIT, for reasons that have yet to be clarified. Known risk factors for developing HIT are tissue trauma and inflammation, since they trigger immune responses and allow antibody production against PF4 [52,53]. The development of thrombotic events following COVID-19 vaccination with mRNA vaccines does not seem to be pathophysiologically different from thrombosis not associated with COVID-19 vaccination. For this reason, thrombotic events observed in a temporal context with mRNA vaccines can be triggered by established risk factors or can occur by chance, but the induction of proinflammatory cascades within the immune response to vaccine administration might contribute as a prothrombotic trigger. Notably, the mechanism behind thrombotic events after mRNA vaccination is different from the pathomechanism of VITT as observed after recombinant adenoviral vector vaccination [36,54,55].

## 5. Diagnostic and Therapeutic Approaches in Vaccine-Induced Thrombotic Thrombocytopenia

Various guidelines and guidance documents have been released in order to provide support for diagnostic and treatment decisions in VITT patients. High-dose intravenous immunoglobulins (IVIG) and anticoagulation represent the main components of the therapeutic management of VITT [56]. According to the first investigation results from VITT patients by Greinacher et al. [2], anticoagulant treatment with non-heparin anticoagulants is recommended, due to pathophysiologic and clinic parallels with autoimmune HIT. Recommendations regarding IVIG administration are based on available evidence regarding the treatment of severe autoimmune HIT [39,57], but also on findings by Greinacher et al. [2] which show effective platelet inhibition in VITT.

By 1 April 2021, the Society of Thrombosis and Haemostasis Research (GTH) published guidance statements on the diagnostic and therapeutic management of VITT patients following ChAdOx1 nCoV-19 vaccination. The diagnostic algorithm included the execution of a screening test for HIT in order to detect antibodies against PF4 in patients developing confirmed thrombosis and/or thrombocytopenia between days four and sixteen after receiving a ChAdOx1 nCoV-19 vaccination. In the case of a positive screening test for antibodies directed against PF4, an immunological cause is likely and, thus, further diagnostic workup and the avoidance of heparins and heparin-like anticoagulants are recommended. A functional confirmatory test should be performed by use of a classical heparin-induced platelet activation assay (HIPA) or otherwise by a serotonin-release assay (SRA). If the HIT screening test is negative since no antibodies against PF4-heparin are detectable, and if the test used is suitable for the detection of these antibodies by appearing to have an appropriate sensitivity for the relevant antibodies, thrombosis in the context of a VITT phenomenon can be safely ruled out. In the case of a positive screening test where there is an inability to confirm the functional effects of the detected antibodies in a classic HIPA or SRA, a modified HIPA assay is recommended [58]. Therapeutic recommendations by the GTH suggested the use of non-heparin anticoagulants, including danaparoid, argatroban, direct oral anticoagulants (DOACs), and possibly fondaparinux, until an autoimmune HIT phenomenon is ruled out. Apart from the anticoagulation aspect, the GTH presents the possibility of using high-dose IVIG, at a dose of 1 g/kg of body weight, daily on two consecutive days in order to interrupt the prothrombotic pathomechanism that drives the VITT phenomenon [58]. Recommendations for IVIG-administration within the GTH guidance were based on the evident effect of rapid inhibition of HIT-antibody induced platelet activation. [57,59] A number of case reports published within the following weeks and months confirmed the safety and efficacy of IVIG-treatment in VITT patients [56,60,61,62]. The GTH guideline document discusses conducting VITT diagnostic prior to administration of IVIG, due to possible false negative tests resulting from IVIG treatment [58].

By the end of April 2021, the International Society on Thrombosis and Hemostasis (ISTH) issued detailed and very feasible interim guidance for the diagnostic and treatment management of VITT. The first step of the diagnostic flow chart contained in this guidance is based on (1) the evaluation of signs and symptoms of thromboembolism and (2) the evaluation of symptom onset in a predefined window following COVID vaccination. In cases where COVID-vaccination with ChAdOx1 nCoV-19 or Ad26.COV2.S 4 was administered up to 28 days prior to the new onset of typical signs and/or symptoms of CVT, splanchnic vein thrombosis, deep vein thrombosis or pulmonary embolism, VITT is possible. As compared to GTH recommendations, the ISTH defined an expanded time window for the appearance of thrombosis signs and symptoms of up to 28 days following vaccinations [63]. Typical signs and symptoms of the most frequently appearing thrombotic events within the VITT phenomenon are summarized in Table 2.

**Figure 1:**
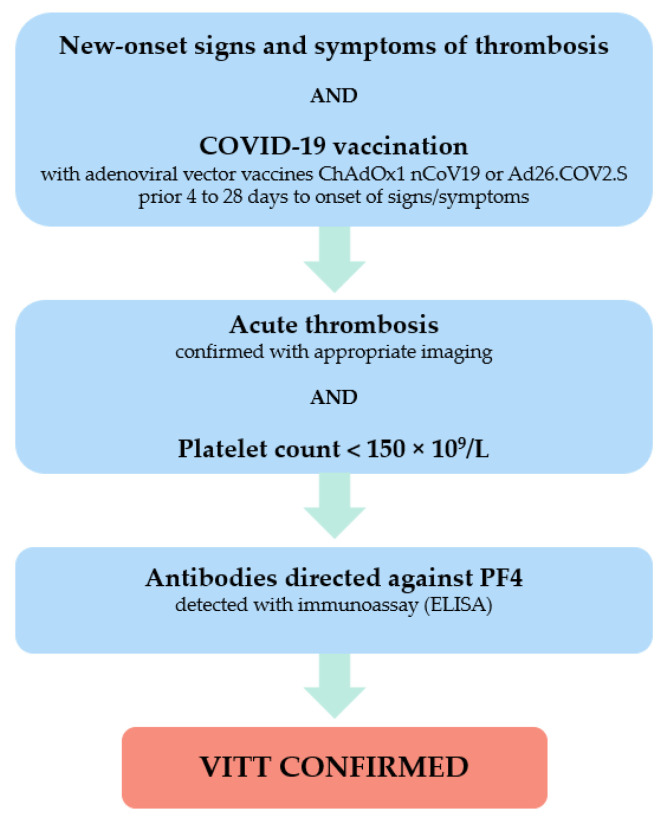
Diagnostic algorithm for vaccine-induced thrombotic thrombocytopenia (modified from ISTH [63]).

In the second step, appropriate imaging in order to confirm the suspicion of thromboembolism, and blood count determination are recommended. The first diagnostic test recommended for suspected CVT is unenhanced computed tomography (CT) to rule out alternative diagnoses in the first step, but more sensitive imaging techniques such as CT venogram or contrast enhanced (magnetic resonance) MR venogram are required to safely rule out CVT [64]. In the case of confirmed thrombosis and thrombocytopenia, as defined by a platelet count below 150 × 10^9^/L, VITT is possible. In the third step, standard coagulation studies, including measurement of prothrombin time (PT), activated partial thromboplastin time (aPTT), fibrinogen and D-dimer, and also an immunoassay to detect antibodies against PF4, are required. The diagnosis of VITT is confirmed if antibodies against PF4 are detectable in patients with an acute thrombotic event and concomitant thrombocytopenia, and prior COVID-19 vaccination with an adenovirus vector vaccine in an appropriate time window [63]. The diagnostic flow chart for use in cases of suspected VITT modified from the ISTH algorithm is presented in Figure 1.

**Figure 2:**
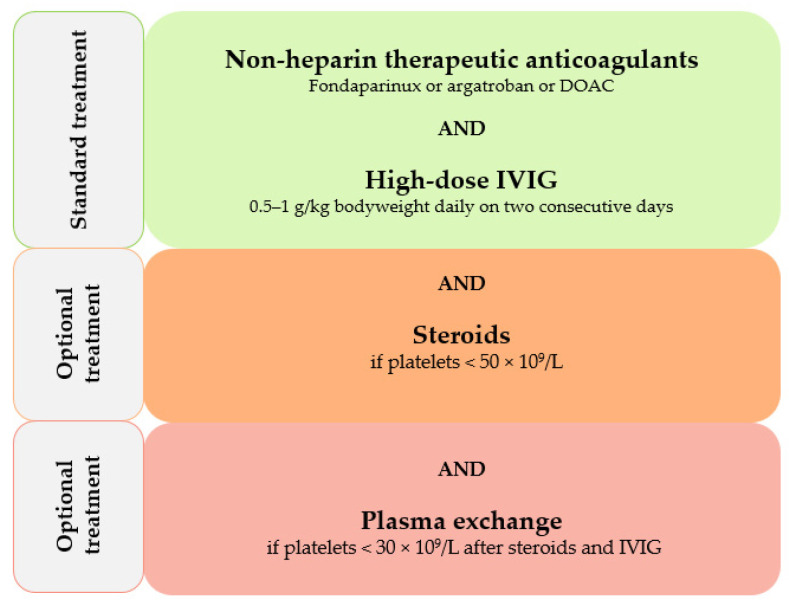
Therapeutic recommendations for vaccine-induced thrombotic thrombocytopenia (modified from ISTH [63]).

Following ISTH guidance, VITT treatment comprises IVIG administration at a dosage of 0.5–1 g/kg daily on two consecutive days, and therapeutic dose anticoagulant treatment with non-heparin anticoagulants. Since detection tests for PF4 antibodies have a slow turnaround time, therapeutic measures should be initiated early in case of a high suspicion of VITT, even before complete test results are available. The preferred options for anticoagulant treatment in VITT patients within this guidance document are argatroban, fondaparinux, and also DOACs in the case of platelets above 50 × 10^9^/L. Additional options are the administration of steroids at a dosage of 1 to 2 mg of prednisone/kg in the case of a platelet count below 50 × 10^9^/L, and also early plasma exchange in the case of a platelet count below 30 × 10^9^/L. If testing for PF4 antibodies is not available, but D-Dimers are 4 times above the upper limit of the normal for exclusion of venous thromboembolism (VTE), patients should be treated as they would in the case of confirmed VITT. Therapeutic measures to be avoided include platelet infusion (except in cases where urgent surgery is required) and also the use of heparins and vitamin K antagonists (VKA) for anticoagulation [63]. Therapeutic options as suggested by the ISTH are shown in Figure 2.

Later on, by July 2021, the WHO issued comprehensive guidance on the diagnosis and management of VITT following COVID-19 vaccination. This guidance clearly recommends ELISA as the preferable method for the detection of antibodies against PF4 in patients with suspected VITT, as rapid immunoassays are not that sensitive. In addition, the WHO defines major and minor criteria for thrombocytopenia, thrombotic events and laboratory examinations for diagnostic workup when VITT is suspected. Following the announced algorithm, a clinical diagnosis of VITT is confirmed in the combined presence of two major criteria of severe thrombocytopenia, as defined by a platelet count below 50 × 10^9^/L and an acute thrombotic event at an uncommon location, such as CVT, splanchnic vein thrombosis, or thrombosis at multiple sites. Confirmation of VITT is also possible in cases of minor thrombosis (located e.g., in pulmonary arteries or veins, limb veins, cerebral arteries, or other arteries/veins) in combination with minor thrombocytopenia (a platelet count below 150 × 10^9^/L but above 50 × 10^9^/L), but with the detection of antibodies against PF4. Regarding therapeutic management options, the WHO guidance document recommends the use of non-heparin anticoagulants (argatroban, bivalirudine, fondaparinux, danaparoid, rivaroxaban, apixaban, dabigatran) and also IVIG (1 g/kg on two consecutive days or 0.4 g/kg on five consecutive days) in individuals with VITT. There is no clear recommendation for the use of steroids stated within this guidance document. Furthermore, the WHO advises against the routine use of platelet infusion in VITT patients [8].

An update on the diagnosis and therapeutic management of VITT patients was issued by Greinacher et al. [64] in October 2021, summarizing treatment options with regard to the likelihood of VITT. Hence, VITT is definitively diagnosed if all of the following five criteria are fulfilled: (1) onset of symptoms five to thirty days following COVID-19 vaccination, (2) verified acute thrombosis or severe persistent headache, (3) platelets below 150 × 10^9^/L, (4) D-Dimer levels more than 4000 fibrinogen equivalent units (and more than eight times the upper limit of the normal), and (5) a positive ELISA assay for antibodies against PF4/heparin. In patients with a confirmed diagnosis of VITT, standard treatment comprises anticoagulation and IVIG administration. If a diagnosis of VITT is not definite because not all of the five criteria are fulfilled, but is probable or possible, anticoagulation and also, in some cases, IVIG treatment, are recommended. Following this classification, anticoagulation might also be indicated without thrombosis but with the fulfilment of other VITT-specific criteria. If VITT is unlikely, anticoagulation is required only in cases where acute thrombosis is detected. Safe anticoagulants for treating acute VITT comprise direct thrombin inhibitors, Factor Xa inhibitors, and fondaparinux. Since VKA causes prothrombotic protein C deficiency, Greinacher et al. advise against the use of VKA in VITT, itself a hypercoagulable state. Aspirin must be avoided since it does not affect platelet activation in the context of VITT, but might increase the risk of bleeding. In addition, Greinacher et al. recommend the use of heparin anticoagulants if non-heparins are not available, since delaying initiation of anticoagulation causes the most severe complications. Steroids might be a reasonable choice if IVIG is not available (e.g., prednisone 1–2 mg/kg or dexamethasone 40 mg over four days). The duration of anticoagulant treatment in VITT patients still remains unclear, but an optimal duration of three to six months, similar to thrombosis without VITT, is assumed [64].

## 6. Prevention of Thrombotic Complications after COVID-19 Vaccination

Following GTH recommendations, thromboprophylaxis in order to prevent the development of thrombotic complications following adenoviral vector vaccines is not routinely indicated, but may be indicated in particular patients with underlying predisposing risk factors for VTE in the case of severe flulike symptoms following vaccination. In case of an indication for thromboprophylaxis following adenoviral vector vaccines, anticoagulants other than LMWH and fondaparinux are advised (e.g., prophylactic dosages of DOACs on an off-label basis). The rationale behind recommending the possible use of therapeutic anticoagulant treatment in VITT, but otherwise advising against its prophylactic use with COVID-19 adenoviral vector vaccines, is the possible activation of inflammatory and immune stimulatory pathways during the early phase after vaccination, which may promote the VITT phenomenon. In patients on pre-existing anticoagulant treatment with oral anticoagulants (OAC), continuation of this OAC is recommended during and after vaccination [58].

## 7. Recommendations for Vaccination in VITT Patients Post-Recovery

Antibodies present in VITT against PF4 are known to decline over time. A study involving 35 VITT patients showed platelet activation assays to become negative within a median of 12 weeks, but full seroconversion, as defined by achieving a negative ELISA, was observed in only three patients. Five patients received a second COVID-19 vaccination with the BNT162b2 vaccine 10 to 18 weeks after their first shot of an adenoviral vector vaccine while still receiving a therapeutic dose anticoagulant treatment, none of them presenting with the appearance of VITT-like changes [65]. Based on the available evidence, the WHO guidance document recommends avoiding repeated application of adenoviral vector vaccines for future vaccination after exploring VITT, and also avoiding adenovirus-based vaccines in patients either with a prior history of HIT or with major thrombosis in their medical history (arterial or venous) and concomitant thrombocytopenia [8]. Even if VITT appears to be almost “a first dose problem” after recombinant adenoviral vector vaccination, Greinacher et al. [64] recommend a preference for mRNA vaccines for further doses in VITT patients. mRNA vaccination appears safe in patients with previous VITT, regardless of whether or not VITT antibodies are still circulating [64].

## 8. Conclusions

Vaccine-induced thrombotic thrombocytopenia following COVID-19 vaccination by use of recombinant adenoviral vector vaccines ChAdOx1 nCoV-19 (AstraZeneca) and Ad26.COV2.2 (Johnson & Johnson/Janssen) is a rare but potentially life-threatening complication. The VITT phenomenon mimics the clinical image of autoimmune HIT by showing strong reactivity on a PF4-heparin enzyme-linked immunosorbent assay (ELISA), due to high-titer immunoglobulin G class antibodies directed against PF4, with enhanced platelet activation in the presence of PF4. In cases of suspected VITT, early diagnosis and treatment initiation without delay are required. High-dose intravenous immunoglobulins (IVIG) and anticoagulation represent the main components of the therapeutic management of VITT. Since VITT closely mimics autoimmune HIT, non-heparin anticoagulants including fondaparinux, thrombin inhibitors, and F.Xa inhibitors are recommended as the first choice treatment, but heparins do not seem to cause severe harm in the majority of VITT patients.

## Figures and Tables

**Table 1:** Overview of vaccine-induced thrombotic-thrombocytopenia (VITT) cases.

Author (First)	Cases (*n*)	Females *n* (%)	Age <60 y *n* (%)	Events (*n*)	Site of Thrombosis				Thrombo- Cytopenia <150 × 10^9^/L	Anti-PF4 Antibodies Positive
					CVT *n* (%)	SVT *n* (%)	VTE *n* (%)	Others *n* (%)		
Greinacher et al. [2]	11	9 (81.8%)	9 (81.8%) *	19 †	9 (47.4)	3 (15.8%)	3 (15.8%)	4 (21.1%)	10 (90.9%) †	9 (81.8%) *
Schultz et al. [3]	5	4 (80%)	5 (100%)	5	4 (80%)	1 (20%)	0 /0%)	0 (0%)	5 (100%)	5 (100%)
Franchini et al. [34]	1	0 (0%)	1 (100%)	1	1 (100%)	0 (0%)	0 (0%)	0 (0%)	1 (100%)	1 (100%)
Scully et al. [4]	23	14 (60.8%)	19 (82.6%)	29	13 (44.8%)	3 (10.3%)	7 (24.1%)	6 (20.7%)	22 (95.7%) †	21 (91.3%) †
See et al. [5]	12	12 (100%)	12 (100%)	25	12 (48%)	2 (8%)	5 (20%)	6 (24%)	12 (100%)	12 (100%)
**Total**	**52**	**39 (75%)**	**46 (88.5%)**	**79**	**39 (49.4%)**	**9 (11.4%)**	**15 (19%)**	**16 (20.3%)**	**50 (96.2%)**	**48 (92.3%)**

* Data are not available for two patients. † Data are not available for one patient. Abbreviations: CVT, cerebral venous thrombosis; SVT, splanchnic vein thrombosis; VTE, venous thromboembolism (pulmonary embolism, lower extremity deep vein thrombosis); PF4, platelet factor 4.

**Table 2:** Signs and symptoms suggestive of venous thrombosis with VITT.

Thrombosis Location	Signs and Symptoms
suggestive of cerebral venous thrombosis	severe persistent headache
	+/− vision change
	+/− seizure-like activity
suggestive of splanchnic vein thrombosis	severe persistent abdominal pain
suggestive of deep vein thrombosis	leg pain and/or swelling
suggestive of pulmonary embolism	chest pain and/or shortness of breath

## Data Availability

Not applicable.

## References

[1] Castells M.C., Phillips E.J. (2021). Maintaining Safety with SARS-CoV-2 Vaccines. N. Engl. J. Med..

[2] Greinacher A., Thiele T., Warkentin T.E., Weisser K., Kyrle P.A., Eichinger S. (2021). Thrombotic Thrombocytopenia after ChAdOx1 nCov-19 Vaccination. N. Engl. J. Med..

[3] Schultz N.H., Sorvoll I.H., Michelsen A.E., Munthe L.A., Lund-Johansen F., Ahlen M.T., Wiedmann M., Aamodt A.H., Skattor T.H., Tjonnfjord G.E. (2021). Thrombosis and Thrombocytopenia after ChAdOx1 nCoV-19 Vaccination. N. Engl. J. Med..

[4] Scully M., Singh D., Lown R., Poles A., Solomon T., Levi M., Goldblatt D., Kotoucek P., Thomas W., Lester W. (2021). Pathologic Antibodies to Platelet Factor 4 after ChAdOx1 nCoV-19 Vaccination. N. Engl. J. Med..

[5] See I., Su J.R., Lale A., Woo E.J., Guh A.Y., Shimabukuro T.T., Streiff M.B., Rao A.K., Wheeler A.P., Beavers S.F. (2021). US Case Reports of Cerebral Venous Sinus Thrombosis With Thrombocytopenia After Ad26.COV2.S Vaccination, March 2 to April 21, 2021. JAMA.

[6] Schulz J.B., Berlit P., Diener H.C., Gerloff C., Greinacher A., Klein C., Petzold G.C., Piccininni M., Poli S., Rohrig R. (2021). COVID-19 Vaccine-Associated Cerebral Venous Thrombosis in Germany. Ann. Neurol..

[7] Greinacher A., Selleng K., Warkentin T.E. (2017). Autoimmune heparin-induced thrombocytopenia. J. Thromb. Haemost..

[8] WHO Guidance for clinical Case Management of Thrombosis with Thrombocytopenia Syndome (TTS) Following Vaccination to Prevent Coronavirus Disease (COVID-19). https://apps.who.int/iris/bitstream/handle/10665/342999/WHO-2019-nCoV-TTS-2021.1-eng.pdf?sequence=1&isAllowed=y.

[9] Ropper A.H., Klein J.P. (2021). Cerebral Venous Thrombosis. N. Engl. J. Med..

[10] Stam J. (2005). Thrombosis of the cerebral veins and sinuses. N. Engl. J. Med..

[11] Warkentin T.E., Greinacher A. (2005). Thrombosis of the cerebral veins and sinuses. N. Engl. J. Med..

[12] Otite F.O., Patel S., Sharma R., Khandwala P., Desai D., Latorre J.G., Akano E.O., Anikpezie N., Izzy S., Malik A.M. (2020). Trends in incidence and epidemiologic characteristics of cerebral venous thrombosis in the United States. Neurology.

[13] Chan B.T.B., Bobos P., Odutayo A., Pai M. (2021). Meta-analysis of risk of vaccine-induced immune thrombocytopenia and thrombosis following ChAdOx1—A recombinant vaccine. MedRxiv.

[14] Spanish Agency of Medicine and Healthcare Products Pharmacovigilance Report, Published on 9 April 2021. https://www.aemps.gob.es/acciones-informativas/boletines-de-la-aemps/boletin-mensual-de-farmacovigilancia.

[15] Center for Disease Control and Prevention. National Center for Immunization & Respiratory Diseases Update: Thrombosis with Thrombocytopenia Syndrome (TTS) Following COVID-19 Vaccination. https://www.cdc.gov/vaccines/ACIP/meetings/downloads/slides-2021-05-12/07-COVID-Shimabukuro-508.pdf.

[16] European Medicines Agency 29 March 2021 Update. COVID-19 Vaccine Safety Update VAXZEVRIA AstraZeneca AB. https://www.ema.europa.eu/en/documents/covid-19-vaccine-safety-update/covid-19-vaccinesafety-update-vaxzevria-previously-covid-19-vaccine-astrazeneca-29-march-2021_en.pdf.

[17] European Medicines Agency (2021). COVID-19 Vaccine Janssen: EMA Finds Possible Link to Very Rare Cases of Unusual Blood Clots with Low Blood Platelets. https://www.ema.europa.eu/en/news/covid-19-vaccine-janssen-ema-finds-possible-link-very-rare-cases-unusual-blood-clots-low-blood.

[18] WHO Coronavirus (COVID-19) Dashboard. https://covid19.who.int/.

[19] Dedicated COVID-19 Vaccination Dashboard. https://www.who.int/emergencies/diseases/novel-coronavirus-2019/covid-19-vaccines.

[20] European Centre for Disease Prevention and Control (ECDC) COVID-19 Vaccine Tracker. https://vaccinetracker.ecdc.europa.eu/public/extensions/covid-19/vaccine-tracker.html#distribution-tab.

[21] Our World In Data. Coronavirus (COVID-19) Vaccinations. https://ourworldindata.org/covid-vaccinations.

[22] Kandimalla R., Chakraborty P., Vallamkondu J., Chaudhary A., Samanta S., Reddy P.H., De Feo V., Dewanjee S. (2021). Counting on COVID-19 Vaccine: Insights into the Current Strategies, Progress and Future Challenges. Biomedicines.

[23] World Health Organization (WHO) Coronovirus Disease (COVID-19): Vaccines. https://www.who.int/news-room/questions-and-answers/item/coronavirus-disease-(covid-19)-vaccines?gclid=Cj0KCQjw-JyUBhCuARIsANUqQ_JQix8FzcHYwqzqQWMmGAlmXs2xWFZ4lCPfRRcGiypTeXHVba33d1EaAkbmEALw_wcB&topicsurvey=v8kj13).

[24] European Medicines Agency (EMA) COVID-19 Vaccines. https://www.ema.europa.eu/en/human-regulatory/overview/public-health-threats/coronavirus-disease-covid-19/treatments-vaccines/covid-19-vaccines.

[25] Pardi N., Hogan M.J., Porter F.W., Weissman D. (2018). mRNA vaccines—A new era in vaccinology. Nat. Rev. Drug Discov..

[26] Teijaro J.R., Farber D.L. (2021). COVID-19 vaccines: Modes of immune activation and future challenges. Nat. Rev. Immunol..

[27] Chakraborty C., Sharma A.R., Bhattacharya M., Lee S.S. (2021). From COVID-19 to Cancer mRNA Vaccines: Moving From Bench to Clinic in the Vaccine Landscape. Front. Immunol..

[28] World Health Organizsation (WHO) The Pfizer BioNTech (BNT162b2) COVID-19 Vaccine: What You Need To Know. https://www.who.int/news-room/feature-stories/detail/who-can-take-the-pfizer-biontech-covid-19--vaccine.

[29] Baden L.R., El Sahly H.M., Essink B., Kotloff K., Frey S., Novak R., Diemert D., Spector S.A., Rouphael N., Creech C.B. (2021). Efficacy and Safety of the mRNA-1273 SARS-CoV-2 Vaccine. N. Engl. J. Med..

[30] Humphreys I.R., Sebastian S. (2018). Novel viral vectors in infectious diseases. Immunology.

[31] Connors M., Graham B.S., Lane H.C., Fauci A.S. (2021). SARS-CoV-2 Vaccines: Much Accomplished, Much to Learn. Ann. Intern. Med..

[32] Kaur S.P., Gupta V. (2020). COVID-19 Vaccine: A comprehensive status report. Virus Res..

[33] World Health Organization (WHO) The Janssen Ad26.COV2.S COVID-19 Vaccine: What You Need To Know. https://www.who.int/news-room/feature-stories/detail/the-j-j-covid-19-vaccine-what-you-need-to-know.

[34] Franchini M., Testa S., Pezzo M., Glingani C., Caruso B., Terenziani I., Pognani C., Bellometti S.A., Castelli G. (2021). Cerebral venous thrombosis and thrombocytopenia post-COVID-19 vaccination. Thromb. Res..

[35] Franchini M., Liumbruno G.M., Pezzo M. (2021). COVID-19 vaccine-associated immune thrombosis and thrombocytopenia (VITT): Diagnostic and therapeutic recommendations for a new syndrome. Eur. J. Haematol..

[36] Krzywicka K., Heldner M.R., Sanchez van Kammen M., van Haaps T., Hiltunen S., Silvis S.M., Levi M., Kremer Hovinga J.A., Jood K., Lindgren E. (2021). Post-SARS-CoV-2-vaccination cerebral venous sinus thrombosis: An analysis of cases notified to the European Medicines Agency. Eur. J. Neurol..

[37] Rosenthal M.A., Rischin D., McArthur G., Ribbons K., Chong B., Fareed J., Toner G., Green M.D., Basser R.L. (2002). Treatment with the novel anti-angiogenic agent PI-88 is associated with immune-mediated thrombocytopenia. Ann. Oncol..

[38] Tardy-Poncet B., Tardy B., Grelac F., Reynaud J., Mismetti P., Bertrand J.C., Guyotat D. (1994). Pentosan polysulfate-induced thrombocytopenia and thrombosis. Am. J. Hematol..

[39] Hwang S.R., Wang Y., Weil E.L., Padmanabhan A., Warkentin T.E., Pruthi R.K. (2021). Cerebral venous sinus thrombosis associated with spontaneous heparin-induced thrombocytopenia syndrome after total knee arthroplasty. Platelets.

[40] Jay R.M., Warkentin T.E. (2008). Fatal heparin-induced thrombocytopenia (HIT) during warfarin thromboprophylaxis following orthopedic surgery: Another example of ‘spontaneous’ HIT?. J. Thromb. Haemost..

[41] Warkentin T.E., Basciano P.A., Knopman J., Bernstein R.A. (2014). Spontaneous heparin-induced thrombocytopenia syndrome: 2 new cases and a proposal for defining this disorder. Blood.

[42] Warkentin T.E., Makris M., Jay R.M., Kelton J.G. (2008). A spontaneous prothrombotic disorder resembling heparin-induced thrombocytopenia. Am. J. Med..

[43] Warkentin T.E., Greinacher A. (2021). Spontaneous HIT syndrome: Knee replacement, infection, and parallels with vaccine-induced immune thrombotic thrombocytopenia. Thromb. Res..

[44] Salih F., Schonborn L., Kohler S., Franke C., Mockel M., Dorner T., Bauknecht H.C., Pille C., Graw J.A., Alonso A. (2021). Vaccine-Induced Thrombocytopenia with Severe Headache. N. Engl. J. Med..

[45] Nazi I., Arnold D.M., Warkentin T.E., Smith J.W., Staibano P., Kelton J.G. (2015). Distinguishing between anti-platelet factor 4/heparin antibodies that can and cannot cause heparin-induced thrombocytopenia. J. Thromb. Haemost..

[46] Padmanabhan A., Jones C.G., Curtis B.R., Bougie D.W., Sullivan M.J., Peswani N., McFarland J.G., Eastwood D., Wang D., Aster R.H. (2016). A Novel PF4-Dependent Platelet Activation Assay Identifies Patients Likely to Have Heparin-Induced Thrombocytopenia/Thrombosis. Chest.

[47] Stone D., Liu Y., Shayakhmetov D., Li Z.Y., Ni S., Lieber A. (2007). Adenovirus-platelet interaction in blood causes virus sequestration to the reticuloendothelial system of the liver. J. Virol..

[48] Othman M., Labelle A., Mazzetti I., Elbatarny H.S., Lillicrap D. (2007). Adenovirus-induced thrombocytopenia: The role of von Willebrand factor and P-selectin in mediating accelerated platelet clearance. Blood.

[49] Lopez Yomayuza C.C., Preissner K.T., Lorenz B., Stieger K. (2019). Optimizing Measurement of Vascular Endothelial Growth Factor in Small Blood Samples of Premature Infants. Sci. Rep..

[50] Rauova L., Zhai L., Kowalska M.A., Arepally G.M., Cines D.B., Poncz M. (2006). Role of platelet surface PF4 antigenic complexes in heparin-induced thrombocytopenia pathogenesis: Diagnostic and therapeutic implications. Blood.

[51] Greinacher A., Selleng K., Palankar R., Wesche J., Handtke S., Wolff M., Aurich K., Lalk M., Methling K., Volker U. (2021). Insights in ChAdOx1 nCoV-19 vaccine-induced immune thrombotic thrombocytopenia. Blood.

[52] Lubenow N., Hinz P., Thomaschewski S., Lietz T., Vogler M., Ladwig A., Junger M., Nauck M., Schellong S., Wander K. (2010). The severity of trauma determines the immune response to PF4/heparin and the frequency of heparin-induced thrombocytopenia. Blood.

[53] Gong T., Liu L., Jiang W., Zhou R. (2020). DAMP-sensing receptors in sterile inflammation and inflammatory diseases. Nat. Rev. Immunol..

[54] Dias L., Soares-Dos-Reis R., Meira J., Ferrao D., Soares P.R., Pastor A., Gama G., Fonseca L., Fagundes V., Carvalho M. (2021). Cerebral Venous Thrombosis after BNT162b2 mRNA SARS-CoV-2 vaccine. J. Stroke Cerebrovasc. Dis..

[55] Polack F.P., Thomas S.J., Kitchin N., Absalon J., Gurtman A., Lockhart S., Perez J.L., Perez Marc G., Moreira E.D., Zerbini C. (2020). Safety and Efficacy of the BNT162b2 mRNA Covid-19 Vaccine. N. Engl. J. Med..

[56] Bourguignon A., Arnold D.M., Warkentin T.E., Smith J.W., Pannu T., Shrum J.M., Al Maqrashi Z.A.A., Shroff A., Lessard M.C., Blais N. (2021). Adjunct Immune Globulin for Vaccine-Induced Immune Thrombotic Thrombocytopenia. N. Engl. J. Med..

[57] Warkentin T.E. (2019). High-dose intravenous immunoglobulin for the treatment and prevention of heparin-induced thrombocytopenia: A review. Expert Rev. Hematol..

[58] Oldenburg J., Klamroth R., Langer F., Albisetti M., von Auer C., Ay C., Korte W., Scharf R.E., Potzsch B., Greinacher A. (2021). Diagnosis and Management of Vaccine-Related Thrombosis following AstraZeneca COVID-19 Vaccination: Guidance Statement from the GTH. Hamostaseologie.

[59] Mohanty E., Nazir S., Sheppard J.I., Forman D.A., Warkentin T.E. (2019). High-dose intravenous immunoglobulin to treat spontaneous heparin-induced thrombocytopenia syndrome. J. Thromb. Haemost..

[60] Graf T., Thiele T., Klingebiel R., Greinacher A., Schabitz W.R., Greeve I. (2021). Immediate high-dose intravenous immunoglobulins followed by direct thrombin-inhibitor treatment is crucial for survival in Sars-Covid-19-adenoviral vector vaccine-induced immune thrombotic thrombocytopenia VITT with cerebral sinus venous and portal vein thrombosis. J. Neurol..

[61] Guetl K., Gary T., Raggam R.B., Schmid J., Wolfler A., Brodmann M. (2021). SARS-CoV-2 vaccine-induced immune thrombotic thrombocytopenia treated with immunoglobulin and argatroban. Lancet.

[62] Gattringer T., Gressenberger P., Gary T., Wolfler A., Kneihsl M., Raggam R.B. (2022). Successful management of vaccine-induced immune thrombotic thrombocytopenia-related cerebral sinus venous thrombosis after ChAdOx1 nCov-19 vaccination. Stroke Vasc. Neurol..

[63] International Society on Thrombosis and Hemostasis (ISTH) Interim Guidance for the Diagnosis and Treatment on Vaccine-Induced Immune Thrombotic Thrombocytopenia. https://cdn.ymaws.com/www.isth.org/resource/resmgr/ISTH_VITT_Guidance_2.pdf.

[64] Greinacher A., Langer F., Makris M., Pai M., Pavord S., Tran H., Warkentin T.E. (2022). Vaccine-induced immune thrombotic thrombocytopenia (VITT): Update on diagnosis and management considering different resources: Response to Comment from Yamada. J. Thromb. Haemost..

[65] Schonborn L., Thiele T., Kaderali L., Greinacher A. (2021). Decline in Pathogenic Antibodies over Time in VITT. N. Engl. J. Med..

